# Acute Renal Impairment in Patients Due to Paracetamol Overdose in the Absence of Hepatic Impairment

**DOI:** 10.7759/cureus.20727

**Published:** 2021-12-27

**Authors:** Zahid Khan, Mohammed Abumedian, Mildred Ibekwe, Khalid Musa, Gideon Mlawa

**Affiliations:** 1 Cardiology and Internal Medicine, Barking, Havering and Redbridge University Hospitals NHS Trust, London, GBR; 2 Cardiology, Royal Free Hospital, London, GBR; 3 Geriatrics and Internal Medicine, Barking, Havering and Redbridge University Hospitals NHS Trust, London, GBR; 4 Internal Medicine and Diabetes and Endocrinology, Barking, Havering and Redbridge University Hospitals NHS Trust, London, GBR

**Keywords:** paracetamol toxicity, : acute kidney injury, s: hepatotoxicity, nac- n acetyl cysteine, acute tubular necrosis (atn)

## Abstract

In general, paracetamol poisoning is associated with hepatotoxicity and very rarely with renal impairment in the absence of significant hepatic impairment. Paracetamol poisoning associated with renal impairment is rare, and it is mostly associated with hepatotoxicity. Most patients with acute renal impairment show a pattern of acute tubular necrosis or injury based on their blood, clinical presentation, and imaging. The level of injury was found to be associated with the dose of paracetamol taken.

We describe a case of a 22-year-old patient presenting to the hospital with abdominal pain, back pain, and two episodes of vomiting after 36 hours of an intentional paracetamol overdose of 60 tablets. His lab results showed raised creatinine levels and C-reactive protein (CRP) despite normal liver function tests. His paracetamol and salicylate levels were not checked on his initial presentation. He was given N-acetyl cysteine (NAC) treatment for paracetamol overdose and had computed tomography of kidneys, ureters, and bladder (CT KUB) the following day, which showed mild, uncomplicated sigmoid diverticula. He was discharged the next day, but was readmitted two days later with severe abdominal pain and worsening renal function. He had an magnetic resonance imaging (MRI) abdomen that showed coronal/axial wedge like areas of relative hypo-intense change in the T2 acquisition. He received intravenous fluids and antibiotics, and his renal function improved. He was discharged home with outpatient follow-up and appeared to be fully recovered.

## Introduction

Paracetamol poisoning is mainly associated with hepatotoxicity, and its association with renal impairment is rare. The level of renal impairment risk is directly related to the dose of paracetamol taken. We describe a case of a 22-year-old patient presenting to the hospital with abdominal pain, back pain, and two episodes of vomiting after 36 hours of an intentional paracetamol overdose of 60 tablets (30 gram). His blood results showed raised creatinine levels and C-reactive protein (CRP) despite normal liver function tests on his initial presentation. However, his paracetamol and salicylate levels were not checked, despite the fact that he presented with a paracetamol overdose. He was given N-acetyl cysteine (NAC) treatment for paracetamol overdose and had a computerized tomography scan of his kidneys, ureters, and bladder (CT KUB) the following day that showed only mild, uncomplicated sigmoid diverticular disease. He was discharged home a day after; however, he was readmitted two days later with severe abdominal pain and worsening renal function. He had magnetic resonance imaging (MRI) of his abdomen and pelvis on the second day of his second admission that showed coronal/axial wedge like areas of relative hypo-intense change in the T2 acquisition in both kidneys. He received intravenous fluids and antibiotics and his renal function improved. He was discharged home with outpatient follow up. Previous case reports have described the rare association of paracetamol overdose with renal failure and some of these patients require haemodialysis in the absence of any hepatic injury. Most patients with acute renal impairment show a pattern of acute tubular necrosis or injury based on their blood, clinical presentation, and imaging. In our case report, this patient had normal liver function tests initially, although he had mildly deranged liver function tests at the time of discharge. The patient in our case report had normal liver function tests initially, but had mildly raised alanine aminotransaminase at the time of discharge.

## Case presentation

A 22-year-old patient with a past medical history of depression and no regular medications, presented to the emergency department with an intentional paracetamol overdose of 60 tablets (30 grams) after ingestion. He initially presented to the emergency department with right iliac fossa and back pain in both renal angles associated with two episodes of vomiting early in the day. His initial blood tests showed mild acute kidney injury (AKI) with raised creatinine and high C-reactive protein, and his arterial blood gas (ABG) showed normal lactate. Unfortunately, his paracetamol and salicylate levels were not checked on his initial presentation when he first presented to the hospital, and he received NAC treatment without getting paracetamol and salicylate levels. He received both NAC infusion and intravenous fluids on admission and patient was discharged home the following day after psychiatric assessment who deemed him to be at very low suicidal risk with further follow up in the community. He had non-contrast CT KUB in view of the right loin and bilateral back pain that did not show any acute pathology apart from uncomplicated sigmoid diverticulosis.

**Figure 1:**
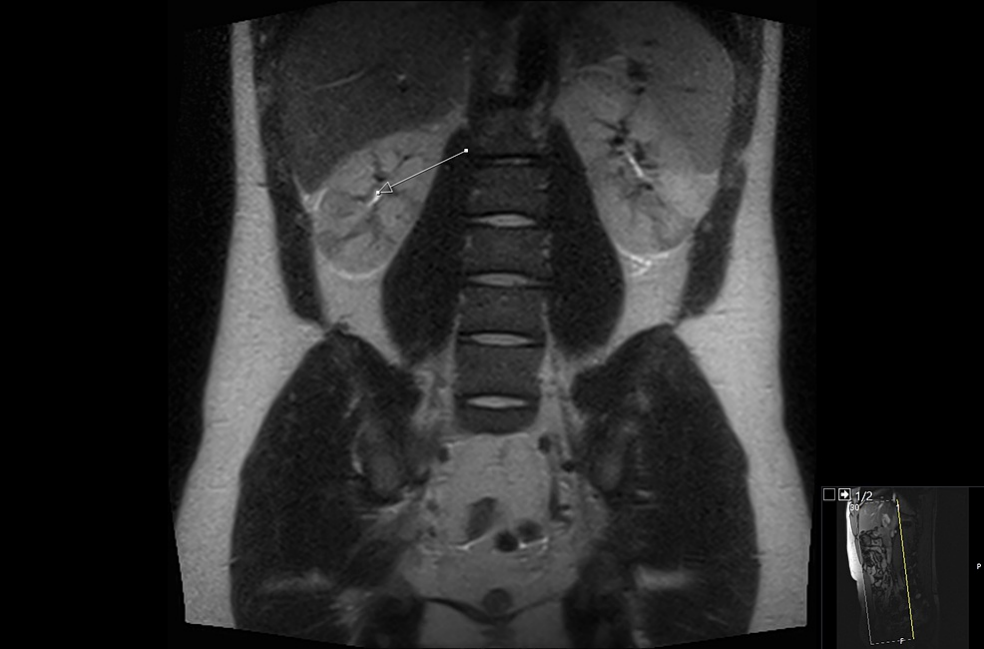
MRI AP showing the T2 acquisition coronal/axial wedge like areas of relative hypo-intense changes in the kidneys, as shown by the pointed arrow.

**Figure 2:**
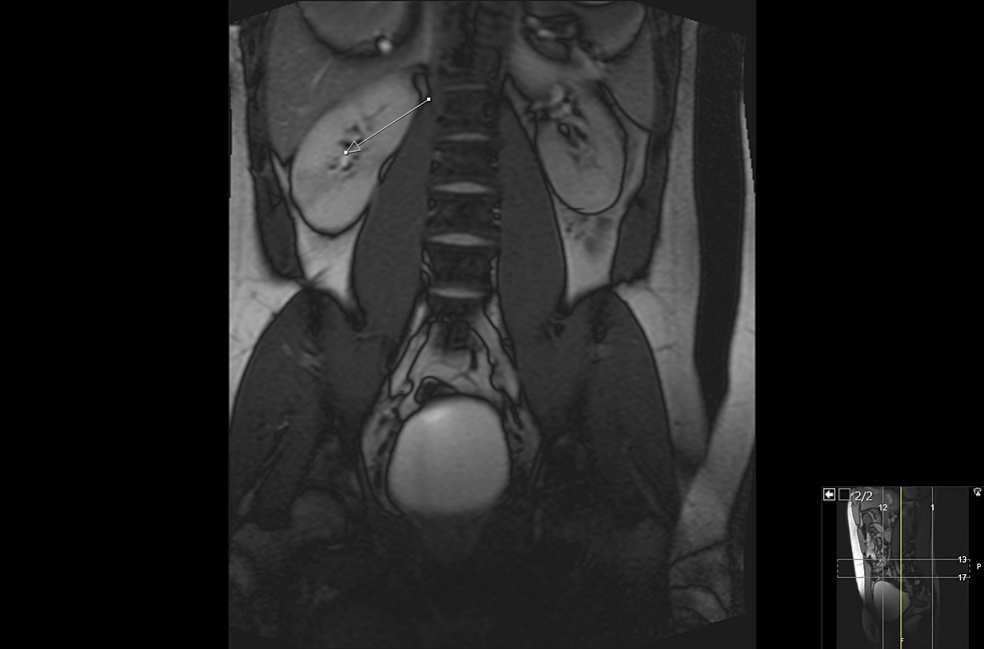
MRI AP showing the T2 acquisition coronal/axial wedge like areas of relative hypo-intensity changes that could represent infarction, infection or inflammation in the area with pointed arrow.

He returned two days later with worsening abdominal and back pain that was not responding to co-codamol. He also had two small episodes of vomiting with rigors and was referred to the surgical team. His blood results showed worsening renal function, with creatinine rising from 126 two days ago to 196 today, and inflammatory markers were also raised, as shown in Table 1. His paracetamol and salicylate levels were less than 1 and 3, respectively, when checked on his second presentation to the hospital. It is important to mention that the levels could be normal as he had received NAC treatment on his first presentation despite his levels not being checked. He had an ultrasound abdomen (US) followed by a non-contrast computerized tomography scan of his kidneys, ureters, and bladder that were reported to be normal. The patient was also given fluids and painkillers, including co-codamol, and was also started on Ibuprofen by the surgical team, and medical review was requested. The patient had received only two doses of Ibuprofen when he was reviewed by the medical team, and his Ibuprofen was stopped. The patient had non-contrast magnetic resonance imaging of the abdomen and pelvis (MRI AP) on the second day of his admission that showed coronal/axial wedge like areas of relative hypo-intense changes in the T2 acquisition in both kidneys that could be due to infarction, inflammation, or infection as shown in Figures 1 and 2. His care was taken over by medical team in view of his worsening renal impairment and no obvious surgical issues.

**Table 1:** Blood results for patient during admission

Blood result	Normal values	Day 1	Days 4	Day 6	Day 8
Haemoglobin	133–173 g/L	147	135	144	151
White cell count	3.8–11 × 10^9^/L	11.8	9.4	8.2	7.9
Neutrophil	2–7.5 × 10^9^/L	9.6	6.9	5.8	4.5
Sodium	133–146 mmol/L	145	140	142	140
Potassium	3.5–5.3 mmol/L	4.7	4.3	4.9	4.7
Urea	2.5–7.8mmol/L	8.1	7.1	6.9	6.0
Creatinine	59–104 μmol	126	196	149	121
Alanine transaminase	0–41 iu/L	21	32	166	108
C-reactive protein	0–5 mg/L	45	61	28	9
Paracetamol level	0–1 mg/L		1		
Salicylate level	0–3 mg/L		<3		

He was reviewed by the pain team in view of his severe pain. His ABG showed high bicarbonate at 34.1, normal lactate at 0.6 mmol/l, and normal pH at 7.35. His midstream specimen of urine (MSU) showed muddy brown casts and his urine dip was positive for blood only. His kidney functions continued to deteriorate, as shown in Table 1. He was reviewed by the renal team, who diagnosed him with acute renal tubular necrosis secondary to paracetamol overdose based on his presentation, imaging, blood and urine results. He was treated with antibiotics and fluids and non-nephrotoxic painkillers such as oxycodone. He started to respond to treatment on day 6 and his kidney functions showed improvement. His renal and autoimmune screens were negative, which was requested on advice of the nephrologist. It is important to mention here that he was not dehydrated as his urea was slightly raised on his first presentation to the hospital, but his lactate was normal. When he presented himself a second time to the hospital, his urea and lactate were both normal, which essentially rules out dehydration as a cause, and clinically he was euvolemic.

He stayed in the hospital for eight days and showed a good physical and biochemical response to treatment, and his blood improved significantly, as shown in Table 1. The patient was discharged home with follow-up in a month's time, and he showed complete resolution of symptoms.

## Discussion

The current report presents a rare phenomenon seen in patients with paracetamol toxicity who present with acute renal impairment in the absence of liver impairment. Previous case reports have described the rare association of paracetamol overdose with renal failure, and some of these patients require haemodialysis in the absence of any hepatic injury [1,2]. A few cases of paracetamol-induced renal injury in the absence of hepatic failure have been reported, and a few of these case reports are in young children younger than 18 years old. Nephrotoxicity with paracetamol without hepatotoxicity is rare and is mostly reversible. The reported incidence of renal toxicity due to paracetamol is 1-2% in most studies, except for a few studies in which it has been reported to be up to 8.9% [3]. There was no obvious predictor for this complication due to paracetamol being found and serial blood tests in children were advised [3].

Another case report of a 15-year-old Vietnamese female who ingested 50-60 325 mg paracetamol tablets and presented to the emergency department 15 hours after the ingestion of the tablets with nausea, mild upper right abdominal and right flank pain. Her paracetamol level was 181 µmol/l and the level was 33 µmol/l following the NAC infusion. Her initial creatinine was 68 µmol/l and her urea was 4.6 mmol/l. Her creatinine began to rise on day 2 and reached 168 µmol/l on day 3 and fell to 90 µmol/l on day 5. Renal impairment due to paracetamol has been described in previous studies and is attributed to acute tubular necrosis both clinically and histologically. The possible explanation for this nephrotoxicity is similar to hepatotoxicity caused by the metabolism of paracetamol by the P450 enzyme system. The patient in our case did not have levels checked due to late presentation (36 hours) to the ED and had a degree of renal impairment on presentation and was treated with NAC. The creatinine level was raised to 196 µmol/l in our patient before its decline [4,5]. One case series reported delayed presentation of renal failure due to significant paracetamol overdose in patients about 48 hours prior to presentation [6,7].

A few published case reports have shown both hepatotoxicity and nephrotoxicity to be present in patients with paracetamol overdose. One such case report is based on the presentation of a 39-year-old patient with schizoaffective and bipolar disorder who took over 100 grams of paracetamol two days prior to his presentation to the emergency department with right upper quadrant pain, nausea, and vomiting. This patient initially had hepatotoxicity due to paracetamol and was treated with NAC. However, this patient started to develop renal impairment on day 3 and, despite improvement in his liver functions, his renal functions continued to deteriorate. His creatinine increased from 0.6 mg/dl on day 1 to 5.7 mg/dl on day 5, and his urea increased from 18 mg/dl to 40 mg/dl on day 5. The likely aetiologies considered were acute tubular necrosis; however, it was excluded as the patient did not have any evidence of hypovolemia or poor perfusion, and this was evidenced by normal blood pressure and normal lactic acid levels in this patient. Hepatorenal syndrome was unlikely as this patient did not have any evidence of hepatic encephalopathy and normal liver function. The most likely aetiology was acute tubular injury secondary to paracetamol. This patient continued to deteriorate and developed confusion and asterixis consistent with uraemia. The patient underwent haemodialysis on days 10 and 11. His renal functions continued to improve thereafter, and his creatinine was 1.32 mg/dl on discharge. The patient in our case report fortunately did not require dialysis and his renal functions showed improvement during admission to treatment [5].

A case series report suggested that renal impairment in patients with paracetamol overdose seems to be unrelated to the degree of liver injury, and NAC treatment did not seem to contribute to the nephrotoxicity. Nine patients had renal dysfunction in this group, and two patients required dialysis who were both comatose, severely hypotensive, and acidotic, and one patient from this group died post-dialysis. The other seven patients showed improvement in their renal functions without dialysis in two to seven days’ time. A total of eight patients received NAC from these nine patients, and a total of six patients received NAC from the group of seven patients who did not require dialysis. They reported that patients with renal impairment tend to be younger and have higher AST levels and lower mean paracetamol levels. The patient in our case report had normal liver function initially, but he developed mild liver impairment over the next two to three days with worsening renal function. However, his renal functions showed improvement on day 7 and continued to improve till his date of discharge from the hospital [3].

The etiology of renal impairment in our patient is most likely due to paracetamol poisoning, as there was no other obvious cause identified for his renal impairment. In addition, his blood results and imaging findings were in keeping with paracetamol-induced acute tubular necrosis. He had normal urea and lactate on his second presentation, and although he was feeling nauseous, he was managing to eat and drink and was not dehydrated. Renal impairment due to paracetamol is due to tubular cell loss, which is a characteristic feature of both acute renal failure and chronic renal disease and is particularly noticeable when apoptosis predominates over mitosis [8]. Paracetamol has been shown to promote hepatocyte apoptosis, and apoptosis generally provides an opportunity for treatment. However, the mechanism for renal cell death and the mode of apoptosis in patients with paracetamol overdose is not clear, and there is evidence that the molecular basis of paracetamol-induced nephrotoxicity may differ from those of hepatotoxicity, as N-acetyl-cysteine protects from the latter, but has been shown not to protect from nephrotoxicity [9,10]. Our patient did not have any urinary symptoms at all and was apyrexial, which makes acute pyelonephritis very unlikely. With larger case series, it may be possible to explore this problem even more and predict the exact incidence of nephrotoxicity alone and combined hepatotoxicity and nephrotoxicity due to paracetamol toxicity.

A case series based on 2068 patients with paracetamol poisoning between March 2005 and October 2007 reported the incidence of acute renal failure due to paracetamol to be only 0.4%, and most patients had hepatotoxicity. Renal failure in these patients was defined by a serum creatinine concentration ≥150 µmol/L (1.69 mg/dL) or ≥50% increase from baseline, and serum creatinine concentrations and alanine aminotransferase (ALT) activity were considered with respect to the interval after paracetamol ingestion. Peak serum ALT activity occurred 2.5 days (2.2-2.9 days) after ingestion, but peak serum creatinine concentrations did not occur until 5.5 days (4.4-5.9 days) after ingestion (p = 0.031 by Wilcoxon test).Renal replacement therapy was not required in these patients, and renal function slowly returned to normal. The authors highlighted, based on this case series, that rising serum creatinine concentrations only became detectable after more than 48 hours after paracetamol ingestion, and therefore, renal failure can easily be missed in these patients if they are discharged home early [7]. Further studies are required to understand the exact prevalence of paracetamol-induced nephropathy alone.

## Conclusions

In conclusion, it is important to remember that paracetamol toxicity can present with acute renal impairment in the absence of liver damage, and these patients should be properly treated to prevent them from developing chronic renal failure. NAC has no role in the management of acute kidney injury in patients due to paracetamol overdose.

Imaging should always be considered in these patients as they tend to present with abdominal pain and other abdominal emergencies that need to be ruled out. Acute tubular necrosis shows good recovery when the underlying insult is corrected and euvolemic status is maintained. It may take one to three weeks for these patients to show complete recovery. It is also important to be aware of the delayed presentation of patients with acute renal failure due to paracetamol overdose, particularly in higher doses, as this can be easily overlooked.
